# 3D Printing Techniques and Their Applications to Organ-on-a-Chip Platforms: A Systematic Review

**DOI:** 10.3390/s21093304

**Published:** 2021-05-10

**Authors:** Violeta Carvalho, Inês Gonçalves, Teresa Lage, Raquel O. Rodrigues, Graça Minas, Senhorinha F. C. F. Teixeira, Ana S. Moita, Takeshi Hori, Hirokazu Kaji, Rui A. Lima

**Affiliations:** 1 MEtRICs, Campus de Azurém, University of Minho, 4800-058 Guimarães, Portugal; inesmaiag@gmail.com; 2 Center for MicroElectromechanical Systems (CMEMS-UMinho), Campus de Azurém, University of Minho, 4800-058 Guimarães, Portugal; teresalage.c@gmail.com (T.L.); raquel.rodrigues@dei.uminho.pt (R.O.R.); gminas@dei.uminho.pt (G.M.); 3 ALGORITMI Center, Campus de Azurém, University of Minho, 4800-058 Guimarães, Portugal; st@dps.uminho.pt; 4 IN+, Center for Innovation, Technology and Policy Research, Instituto Superior Técnico, Universidade de Lisboa, Av. Rovisco Pais, 1049-001 Lisboa, Portugal; anamoita@tecnico.ulisboa.pt; 5 CINAMIL, Department of Exact Sciences and Engineering, Portuguese Military Academy, R. Gomes Freire 203, 1169-203 Lisboa, Portugal; 6 Department of Finemechanics, Graduate School of Engineering, Tohoku University, Sendai 980-8579, Japan; takeshi.hori.b5@tohoku.ac.jp (T.H.); hirokazu.kaji.d4@tohoku.ac.jp (H.K.); 7 Department of Biomedical Engineering, Graduate School of Biomedical Engineering, Tohoku University, Sendai 980-8579, Japan; 8 CEFT, Faculty of Engineering of the University of Porto (FEUP), R. Dr. Roberto Frias, 4200-465 Porto, Portugal

**Keywords:** biofabrication, organ-on-a-chip, 3D bioprinting, in vitro models, biosensors, biomicrofluidics

## Abstract

Three-dimensional (3D) in vitro models, such as organ-on-a-chip platforms, are an emerging and effective technology that allows the replication of the function of tissues and organs, bridging the gap amid the conventional models based on planar cell cultures or animals and the complex human system. Hence, they have been increasingly used for biomedical research, such as drug discovery and personalized healthcare. A promising strategy for their fabrication is 3D printing, a layer-by-layer fabrication process that allows the construction of complex 3D structures. In contrast, 3D bioprinting, an evolving biofabrication method, focuses on the accurate deposition of hydrogel bioinks loaded with cells to construct tissue-engineered structures. The purpose of the present work is to conduct a systematic review (SR) of the published literature, according to the guidelines of the Preferred Reporting Items for Systematic Reviews and Meta-Analyses, providing a source of information on the evolution of organ-on-a-chip platforms obtained resorting to 3D printing and bioprinting techniques. In the literature search, PubMed, Scopus, and ScienceDirect databases were used, and two authors independently performed the search, study selection, and data extraction. The goal of this SR is to highlight the importance and advantages of using 3D printing techniques in obtaining organ-on-a-chip platforms, and also to identify potential gaps and future perspectives in this research field. Additionally, challenges in integrating sensors in organs-on-chip platforms are briefly investigated and discussed.

## 1. Introduction

Drug discovery has been affected adversely by extensive timelines and poor predictive results of preclinical studies, causing an increase in the development costs. The low success rate and the high expense in the later development stage show that there is an urgent need to develop preclinical drug testing models able to predict drug efficacy and safety more accurately, instead of using animal or simpler in vitro models [1,2,3].

Animal models offer a complex physiological structure analogous to the human cell and organ systems. Nevertheless, due to interspecies differences, they are not necessarily predictive of results in humans [4,5]. These models fail to adequately predict and represent human metabolism due to different pharmacokinetics, pharmacodynamics, and metabolic pathways for test substances [6]. Moreover, they are expensive and limited in usability, feasibility, and ethical issues [7]. To overpass the ethical concerns associated, in vitro models have emerged aiming to fulfill the limitation of animal studies in predicting clinical outcomes. In addition, they have become important for drug studies and disease models according to the “3R’s” animal principle (Refine, Reduce, and Replace animal testing) [5]. However, in vitro methods, such as traditional 2D cultures, fail to either recapitulate the 3D microenvironment of in vivo tissues or human physiology [4,8,9]. For this reason, 3D cell-culture models, namely organ-on-a-chip (OoC) platforms, have recently gained great attention since they can be generated with heterogeneous human cells, allowing them to mimic the mechanical, physiological, and chemical characteristics of in vivo tissues. Moreover, their simplicity and possibility of high-throughput screening make this approach unique [9,10]. Thereby, it provides a promising solution to overcome the shortcomings of both conventional 2D cell culture and animal testing [9,11,12,13,14]. 

Over the last decades, conventional manufacturing techniques, such as photo-patterning, self-assembly, and soft lithography, have been used for the manufacturing of microfluidic devices and also OoC. In particular, lithography has been the most common technique performed. Nevertheless, this is a highly expensive process with complicated and time-consuming manual procedures performed in a clean-room environment [10,15,16,17,18,19]. In this sense, recent advances in microfabrication techniques, cell biology, microfluidics, and 3D printing enabled the rapid manufacturing of OoC along with biomimetic tissue micro-architectures, which can provide the basis for preclinical assays with greater predictive power [5]. Compared to conventional manufacturing techniques, 3D printing includes the advantages of unlimited design space, freedom of complex geometries, and reduction of waste products [20,21,22]. In particular, 3D bioprinting is an innovative and promising biofabrication strategy that has played an important role since it allows the deposition of biomaterial-encapsulated living cells in the manufacturing of complex 3D structures with high precision, high accuracy, and high throughput [23,24,25,26,27,28]. Considering those characteristics, bioprinters are expected to establish systems that mimic the microenvironment of the human body in a more appropriate way than the animal models and current 2D cell culture environments, enhancing the accuracy of the results and the clinical usage of OoC [25,29,30]. 

**Figure 1:**
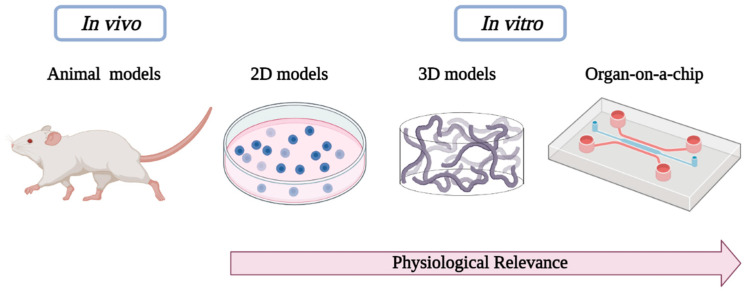
Schematic diagram showing the preclinical models used in biomedical research.

The aforementioned preclinical models used in biomedical investigation are outlined in Figure 1, highlighting the evolution of cell-culture models from simple two-dimensional to complex OoC platforms with three-dimensional bioprinted models.

This paper offers an SR of the literature published in the scope of 3D printing and bioprinting techniques for producing physiologically relevant OoC devices. Additionally, this work aims to discuss the benefits and limitations of using 3D printing techniques to obtain 3D in vitro models, and also to identify potential shortcomings in this research field and suggest directions for upcoming research.

## 2. Methods 

This work was performed in accordance with the Preferred Reporting Items for Systematic Reviews and Meta-Analyses (PRISMA) guidelines for reporting meta-analyses and systematic reviews [31].

### 2.1. Literature Search

The authors systematically and comprehensively saved all studies that used 3D printing techniques to obtain organoids and OoC platforms for in vitro studies from the PubMed, Scopus, and Science Direct databases, using the following keywords combined with the Boolean operators AND and OR: “biofabrication”, “bioprinting”, “rapid prototyping”, “3D printing”, “additive manufacturing”, and “organ-on-a-chip”.

### 2.2. Eligibility Criteria

To perform the study selection, several eligibility criteria were taken into account, namely, inclusion and exclusion criteria. The inclusion criteria consisted of (1) articles published between 2010 and 2020; (2) studies that apply 3D printing and/or bioprinting approaches in the context of OoC devices; and (3) full-text articles. The exclusion criteria comprised (1) abstracts; (2) non-English language literature; (3) reviews, systematic reviews, or meta-analyses; (4) book chapters; (5) short communications; (6) conference articles; (7) patents; (8) case reports; and (9) studies in which fundamental information about the manufacturing process was missing.

### 2.3. Data Analysis

After the articles’ selection from the databases, they were organized in an Excel sheet, from where the duplicates were removed. Then, two authors independently reviewed all the abstracts of the articles, and from those, the same authors selected a set of papers, from which the full texts were again independently read, and a final selection of relevant studies was made. 

After carefully choosing the literature, the results of both authors were compared and discussed until an agreement was reached. 

## 3. Results and Discussion

**Figure 2:**
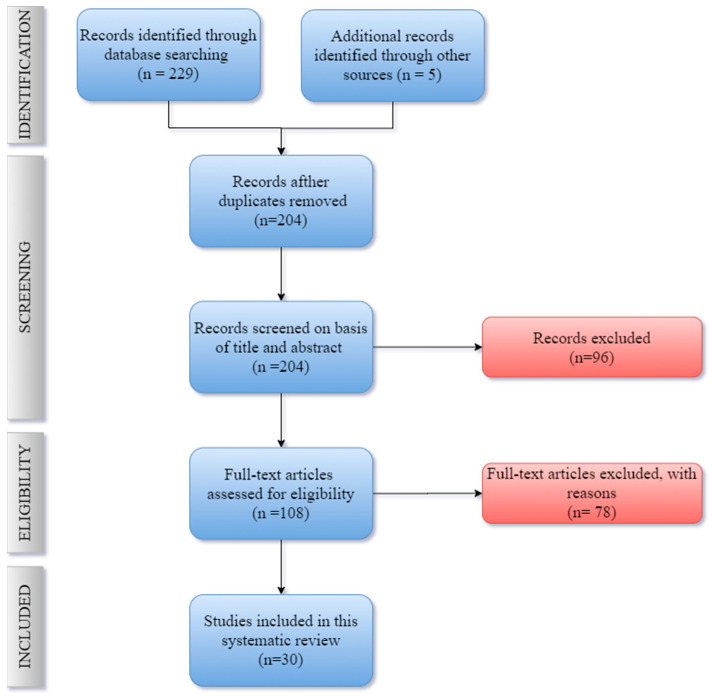
PRISMA flow diagram displaying the procedure of study selection.

**Figure 3:**
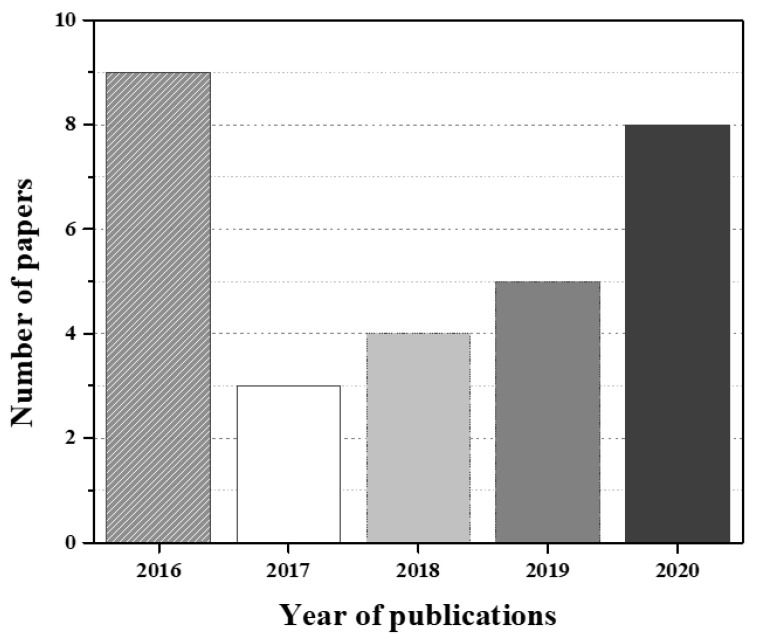
Number of papers and the respective year of publication included in the SR.

As previously mentioned, the authors followed the PRISMA recommended guidance to conduct systematic reviews. Based on the title, 233 potentially relevant articles were identified from the three databases selected. In total, 204 studies were included after removing duplicates. After the evaluation of abstracts, 96 articles were dismissed due to lack of data and different study strategies; thus, 108 full papers were analyzed. In the end, a total of 30 full-text articles were selected. Figure 2 explains the PRISMA flow chart for the selection process of studies incorporated in this systematic review, and Figure 3 shows the number of papers and the respective year of publication of the selected studies.

Among the included studies, it was found that the years of publication have varied between 2016 and 2020, and 75% correspond to studies that have addressed the fabrication of OoC devices through 3D bioprinting technologies, while 25% represent studies that have used 3D printing techniques, which by itself shows the preferred technique to fabricate OoC. 

In the present search, different 3D (bio)printing techniques, cells, and bioinks were found. Thus, to better organize the gathered information, the results are presented in three sections, which include studies that addressed 3D printing and bioprinting techniques applied to specific organ models, and other innovative techniques used to obtain OoC platforms without specifying the target organ, respectively. 

### 3.1. 3D Printing Techniques

3D printing has become a growing field in different areas and it has gained great interest, because of the ability to build complex structures through a layer-by-layer process with different materials in an affordable way [30]. For these reasons, some authors have been using 3D printing techniques to rapidly fabricate the microfluidic models and holders for OoC devices (Table 1).

Looking at the previous results, it can be observed that only 7 (out of 30) papers were identified for this systematic review using 3D printing techniques. Despite the low number of papers identified, it can be seen that 3D printing techniques are mainly used to obtain molds for the devices’ fabrication. Moreover, it can be also observed that distinct 3D printing techniques were selected practically for the same goal. The identified technologies comprised extrusion-based technologies (FDM); inkjet 3D printing and powder bed, which includes plaster-based printing; and light polymerized 3D printing (SLA and DLP).

The extrusion-based technology is the most common and probably the simplest and most affordable 3D printing technique in which a coil of thermoplastic filament feeds into an extrusion head, heated above the melting temperature of the material, and then the material is extruded and solidified [37]. 

Plaster-based printing or inkjet printing involves the printing of jet-ink materials comprising low-viscosity droplets in consecutive layers, and complex shapes in polymer, metal, and ceramic objects can be printed. Once the material is solidified, the printing plate is lowered, and the next layer is printed [38].

Regarding the light polymerized 3D printing techniques, SLA and DLP were used. In SLA, a photopolymer is cured by an ultraviolet laser. Once the curing of the initial layer of the liquid resin is completed, the build platform is lowered, so a new layer of liquid can cover the now-solid planar sections. Conversely, DLP is an emergent technology in which a digital micromirror device, an array of up to several millions of micromirrors, can be rotated independently to modulate the UV light and project an optical pattern [37].

For instance, Nie et al. [32] printed a set of molds with diverse forms of channels in a spiral, serpentine, and multilayer forms to pour hydrogel formulations, and showed that when collagen-treated channels were seeded with HUVECs, they aligned by themselves and lumen structures started to form, indicating the vascularization of the channels. Another similar study was led by Khalid et al. [33], in which a lung cancer-on-chip device with multi-sensors for trans-epithelial electrical impedance-based cytotoxicity assessment of drug candidates was devised. By means of an inkjet printer, an elastomeric microfluidic channel (MC) was printed onto a transparent ITO-patterned glass kept together by a 3D-printed chip support. On the inferior side of the upper glass, the MC was printed, whereas the microfluidic connectors were attached on the higher side of the upper glass to deliver external fluidic routings. Overall, the lung cancer-on-chip platform allowed to perform real-time physiological monitoring using integrated biosensors, making this a promising tool for the cytotoxicity evaluation of novel drug compounds and the improvement and development of personalized medicine. Soker and co-workers [4] devised a metastasis-on-a-chip consisting of two compartments in which liver and gut constructs are housed autonomously, but connected in series through circulating fluid flow. For this purpose, the research team produced molds with 3D printing and they were used to cast with PDMS. After that, hydrogel tissue constructs were formed and cultured in each of the device chambers. This study brings important findings for cancer treatments by mimicking the phenomena of metastasis—the translocation of metastatic tumor cells located on a primary tissue site to a downstream tissue site.

Another different approach was proposed by Ozbolat et al. [15]. The research team, initially, prepared a PDMS mixture and poured it into a petri dish. Following the curing process, using a silicone elastomer ink, several rectangular frames were 3D printed over the cured PDMS. Then, channel prototypes were printed inside of the frames utilizing Carbopol ink. The Slygard 184 blend was then dispensed over the Carbopol prototypes to overlay it. To finish, by washing with deionized water and, subsequently, with Dulbecco’s phosphate-buffered saline solution, the sacrificial Carbopol prototype was detached from each device. To validate the efficiency of the manufactured devices, the channels were aligned with HUVECs and human bone marrow endothelial cells in separate devices, and both demonstrated the growth of endothelium with cells lined in the same way of the fluid flow.

The aforementioned studies used 3D printing techniques without any surface treatment of the printed materials; nevertheless, some authors have explored this theme. For instance, Cho et al. [34] fabricated a PDMS-based OoC by casting a 3D-printed template. However, since the 3D printing resolution is not enough for promoting cellular adhesion, the authors used a cutter machine to create the patterns on the inner channel surfaces of the OoC. This technique can provide a textural detail that may improve, more simply and affordably, the adhesion of the cells onto the OoC’s substrates. In a more recent study, Lantada and co-workers [35] presented an innovative and wide-ranging fabrication method in which laser stereolithography 3D printing, laser material processing on microscale, micro-injection molding, and electroforming were used to fabricate a multi-OoC. Firstly, the master pieces were manufactured using a laser stereolithography machine. Secondly, micro-gates were generated by laser ablation, in the walls that separate the different channels. Thirdly, the master mold was glued onto a thick copper substrate and an electroforming process was done. This process allows obtaining a rigid and homogenous metal block with a uniform thickness, which is fundamental for the final step—the injection molding process, used for creating the final OoC device in an industrial production way. With the injection molding process, the replication of highly detailed microstructures with outstanding surface quality can be obtained. In this situation, the authors used PMMA as the molding material. The authors stated that this methodology allows the industrialized mass production of OoC using thermoplastics suitable for in vitro testing with improved biomimetic performance.

Shrestha and co-workers [36] presented a protocol for surface modification of 3D-printed molds by DLP, in order to fabricate a lung-on-a-chip with high resolution by using PDMS casting. Initially, the 3D-printed mold is washed with isopropanol and then, after the print, by high-pressure air drying. Following that, the face of the cast is prepared to be easily detached from the PDMS. Hence, oxygen plasma treatment was carried out. In the last step, the face of the cast was silanized using trichlorosilane. By testing different membranes and extracellular matrix (ECM) coatings, the manufactured chip was further optimized for extended viability and cell growth. In general, with this methodology, OoC platforms can be rapidly fabricated and the molds can also be utilized for repeated PDMS casting.

Despite the differences between the abovementioned technologies, all authors concluded that the usage of 3D printing techniques to fabricate OoC is simple, cost-effective, robust, and allows the mass manufacture of customized OoC devices. However, attention must be taken in the selection of the 3D printing technique to obtain molds for PDMS casting. For example, molds printed via SLA/DLP methods may not be appropriate for PDMS casting because residual oligomers and monomers on the top of the 3D-printed pieces hamper PDMS polymerization [36]. Hence, the development of optimized surface treatments is crucial for ensuring long-term cell viability in OoC devices. Furthermore, the material utilized in 3D printing must be selected taking into account the curing temperature of the casting material in order to prevent material strain and microstructure deformation, which consequently can affect the cell viability.

### 3.2. 3D Bioprinting Techniques

As previously stated, 3D bioprinting can be described as the spatial distribution in a defined pattern of living cells. The cells are loaded and assembled through layer-by-layer deposition methods assisted by means of a computer, and used for the manufacture of organ analogs and living tissue for a different set of applications, such as pharmacokinetic, tissue engineering, cancer research, and regenerative medicine, among others [39]. For this purpose, biocompatible materials, such as alginate, gellan-gum, collagen, fibrin, and gelatin, are usually used to form hydrogels, called bioinks, to encapsulate cells (cf. Table 2) in order to protect them during the printing process.

Because of its ability to generate complex heterocellular structures with precision at the anatomical level, this manufacturing technique has made possible the convenient fabrication of micro-tissues in OoC platforms [20,30]. Table 2 shows the research works selected that used 3D bioprinting approaches to obtain specific OoC devices, which are discussed below.

Taking into account the studies presented in this section, it can be seen that 3D printing techniques are versatile and they can be applied to obtain a variety of OoC or multi-organ-on-a-chip, such as nervous-system-on-a chip, vascularized tissue-on-a-chip, liver-on-a-chip, renal tubule-on-a-chip, vessel-on-a-chip, myocardium-on-a-chip, gut-on-a-chip, thrombosis-on-a-chip, and tumor array-on-a-chip. These different cases are now presented.

#### 3.2.1. Nervous-System-on-a-Chip

The development of OoC platforms that represent the nervous system is vital since the treatment of neurological disorders is a critical medical challenge. In this sense, Johnson et al. [40] displayed the potential use of extrusion-based 3D cell printing. They dispensed PCL onto the culture dish to create microchannels and deposited grease and silicone across the channels to build compartmentalized chambers. Afterward, they dispensed four-cell suspensions into the different chambers and showed the formation of an interconnected nervous system, proving that this is an effective fabrication approach to help the development of customizable OoC technologies. 

The Bowser research group [41] also fabricated a central nervous system-on-a-chip; however, they did this by means of magnetic bioprinting. Firstly, spinal cord spheroids were manufactured using magnetic nanoparticles, and then they were bioprinted on the hydrogel constructs using their magnetic properties to control placement. Furthermore, a digital projection lithography setup was used to pattern the hydrogel, improving construct uniformity and providing a 3D culture setting holding a macrostructure that supports and guides long-distance 3D projections from neurons. This study conveys important findings regarding the merge of a spheroid, hydrogel culture, and bioprinting as an alternative to more expensive and specialized processes.

#### 3.2.2. Multi-Organ-on-a-Chip

Similarly, by 3D extrusion bioprinting, Skardal and colleagues [42] developed a three-tissue OoC system, comprised of lung, liver, and heart integrated with tissue organoids obtained with customized fluidic device technologies and tissue-specific bioinks. Cardiac and hepatic components were bioprinted into spherical organoids inside customized hydrogel constructs that were placed inside the microreactor devices, while lung modules were generated by forming layers of cells on top of porous membranes incorporated in microfluidic devices. Microreactors with cardiac organoids and liver organoids were connected in series with lung modules, as presented in Table 2. The authors noted drug responses that were dependent on inter-tissue interaction, demonstrating the worth of multiple tissue combinations for in vitro evaluations of both side effects and efficiency associated with new candidate drugs.

#### 3.2.3. Vascularized Tissue-on-a-Chip

Kolesky et al. [43] created a multi-material 3D extrusion bioprinting method that allows the formation of thick human tissues (>1 cm) with multiple cell types, embedded vasculature, and an engineered ECM. Moreover, it is possible to actively perfuse the 3D vascularized tissues for long time periods. The aptitude to perfuse and construct 3D tissues that integrate endothelium, stroma, and parenchyma is an opening step toward making human tissues for in vivo and ex vivo applications.

#### 3.2.4. Liver-on-a-Chip

The liver is of major importance in numerous fundamental functions to preserve normal physiological activities. However, injury instigated by adverse reactions to drugs and chronic diseases may prejudice its ability to perform physiological functions [56]. Bhise and the research team [44] developed a liver-on-a-chip with the HepG2/C3A spheroids directly 3D bioprinted within the culture chamber of a bioreactor for drug toxicity evaluation, using a direct-write 3D bioprinter. The authors verified that the cultured spheroids remained functional during the thirty days of the culture period. Besides, the effects reported on the liver-on-a-chip device to acute acetaminophen were similar to in vitro and animal models, making this a good candidate for drug toxicity investigation. Furthermore, the proposed concept of a bioreactor interfaced with bioprinters allows the realization of innovative generation OoC platforms.

On the other hand, Lee and co-workers [10] proposed a new 3D bioprinting method in a simple one-step fabrication process that can be incorporated into various OoC platforms. However, they focused on liver-on-a-chip since this model plays an important part in the drug discovery process. For this purpose, an in-house extrusion printing system with multiple heads was used. In their study, instead of PDMS, a 3D-printed platform of poly(ε-caprolactone)—PCL—was used where ECM-based hydrogels were placed. The entire chip device was produced by alternatively dispensing the PCL and the two bioink-holding cells. They noted that protein absorption on the printed platform was very small, ensuring accurate measurement of drug sensitivity and metabolism. Furthermore, heterotypic cell types and biomaterials were placed at the desired position, which allows mimicking the natural conditions of the organs. Actually, liver function was found to be significantly improved on the 3D-bioprinted liver-on-a-chip. Nonetheless, the PCL material, when compared to PDMS, shows inferior optical transparency, which has to be overcome. More recently, the same authors [45], using the same printing process, designed a 3D liver-on-a-chip, incorporating a co-culture of multiple cell types. The bioink used liver decellularized ECM and, together with hepatic cells, formed the 3D microenvironment that also included a vascular/biliary fluidic channel for producing vascular and biliary systems, which are fundamental for bile acid removal. However, in this study, poly(ethylene/vinyl acetate) (PEVA) served as the housing material for the OoC and was printed on transparent sterilized PMMA plates. Afterward, gelatin and hepatic dECM bioinks were arranged and used for cell printing. The 3D cell-printed liver-on-a-chip comprised two fluidic channels, a biliary channel on the bottom, and a vascular channel above. In general, the authors developed an advanced liver-on-a-chip, which presented with the biliary system incorporated, and obtained good results with OoC, presenting an efficient drug response. Recently, this research group [46] further deepened the development of liver-on-a-chip platforms, but this time considering liver fibrosis, which can lead to liver cirrhosis, cancer, or liver failure. The OoC platform was developed containing 3 liver cell types (endothelial cells, activated stellate cells and hepatocytes) by their new cell-printing technique in which each nonparenchymal hepatic cell type is delivered in ways of a multilayer construct by using gelatin bioinks. So, the final fibrosis-on-a-chip exhibited characteristics of liver fibrosis, such as collagen accumulation, cell apoptosis, and reduced liver functions, showing/revealing to be a promising in vitro test device for drug discovery. Note that the bioinks used in these studies consisted of gelatin and dECM liver bioinks. The gelatin bioink is in the gel state at a low temperature, while at 37 °C it turns into a liquid state. Therefore, after cell-printing and incubation, the gelatin material in the liquid state was removed, and only the cell component remains. Alternatively, the liver dECM bioink is used to encapsulate hepatocytes and after the incubation process, it provides a 3D environment with the appropriate liver ECM components.

#### 3.2.5. Renal Tubule-on-a-Chip

The development of kidney models is also important since it allows to better understand pathological renal physiology, in predicting nephrotoxicity, and in advancing the treatment of chronic renal diseases. Homan and colleagues [47] designed in vitro 3D human renal proximal tubules (PT) fully incorporated within an ECM and held in perfusable tissue chips. Firstly, a silicone gasket is printed on a glass, and a sheet of engineered ECM constituted of a gelatin–fibrin hydrogel is then deposited inside the gasket. Afterward, a fugitive ink of Pluronic is printed on the upper part of the ECM layer, which will be liquefied and detached from the last 3D PT construct. Following printing, hollow metal pins interfaced across the gasket walls are connected to the fugitive ink and supplementary ECM is cast on the printed structure. Finally, cell media passes through the 3D intricate tubular architecture on-chip by using a peristaltic pump externally placed.

So, briefly, the 3D convoluted PTs comprise an open lumen architecture with PT epithelial cells (PTECs), incorporated into an ECM, and housed within a perfusable tissue chip, where they undergo physiological shear stresses, which exhibited significantly enhanced functional properties and epithelial morphology than the 2D cultures.

#### 3.2.6. Vessel-on-a-Chip

Vascular networks are present in almost all human tissues as the fundamental unit of oxygen, nutrient, waste, and signaling molecule transport; so, its study is of substantial importance. Gao and co-workers [48] fabricated 3D vessel-like structures to be integrated into OoC devices to simulate the microenvironment of blood vessels. For this purpose, 3D hydrogel-based vascular structures were fabricated incorporating multi-level fluidic channels (macrochannels for mechanical stimulation, microchannels for chemical stimulation and nutrient delivery) by using coaxial nozzle-assisted extrusion-based bioprinting, with two nozzles. Briefly, this fabrication process is grounded on the fusion of hollow alginate filaments with smooth muscle cells and fibroblasts printed around a rod template, and endothelial cells are seeded into the inner wall. Then, due to the union of adjacent hollow filaments, two-level fluidic channels are generated: the micro-channel around the wall and a macrochannel in the middle. The results showed that the fibroblasts in the fabricated structures maintained a high viability after being cultured in culture media for one week, proving the good biocompatibility of this method.

From a different perspective, Abudupataer et al. [49] used a light-based 3D bioprinting technology to print 3D constructs containing smooth muscle cells and endothelial cells on a microfluidic chip. Using this novel technology, vascular-related tissues in vitro were produced and provided the technical basis for the construction of vascular disease models and further applications for drug screening. After printing the cell-laden hydrogels on the microfluidic chip, a continuous flow of the medium was perfused in the channel of the chip to establish a vessel-on-a-chip model, mimicking blood flow in the vessel.

The proposed model presents different vascular cell types and biomaterials at the desired position and it provides hydrodynamic and mechanical properties, which create a more realistic vascular tissue in vitro.

#### 3.2.7. Myocardium-on-a-Chip

Mimicking the native structure of the cardiac tissue by 3D bioprinting is an intricate and challenging task, greatly limited by the choice of inks. Zhang et al. [50] proposed a novel strategy grounded on 3D bioprinting, for the manufacturing of endothelialized myocardium-on-a-chip. They used a commercial bioprinter together with a personalized coaxial nozzle mounted from syringe needles. The bioink, made of a combination of photoinitiator, alginate, and GelMA, Irgacure^®^, was fed by an internal needle, while a crosslinking solution of CaCl_2_ was simultaneously dispensed using another needle. When the two fluids come into contact at the tip of the printhead, the formation of microfibers and their deposition in the 3D space is performed. Subsequently, endothelial cells were directly printed inside the microfibrous hydrogel scaffolds, gradually migrating towards the peripheries and forming a sheet of confluent endothelium. This was enabled by the dual-step crosslinking procedure of the composite bioink developed. Then, cardiomyocytes were incorporated on the 3D endothelial layer to create an aligned myocardium able to have synchronous and spontaneous contraction. Additionally, a microfluidic perfusion bioreactor was designed to engineer the endothelialized heart-on-a-chip platform for cardiovascular drug screening.

Recently, Mehrotra et al. [51] developed an innovative biomaterial-ink based on non-mulberry silk fibroin (nSF) protein. n-SF protein is mechanically robust and can be utilized as an ink component in order to improve the mechanical strength of inks, while maintaining their unique physical and biological properties. The proposed ink showed exceptional properties, by allowing not only the fabrication of anisotropic cardiac constructs, which exhibited an elastic behavior similar to the native heart tissue, but also promoted the maturation, maintenance of the cytoskeletal structure, and beating potential of the cardiomyocytes. Moreover, by combing the cardiac constructs with the microfluidic perfusion-based bioreactor, the vascularized myocardial tissue-on-a-chip model can be utilized as a potential platform for screening several drugs.

#### 3.2.8. Gut-on-a-Chip

Another vital organ that is also very complex and difficult to mimic is the human intestine since it shows an anatomically complex architecture. Kim and the research team [52] designed a 3D intestinal villi prototype comprising a microvascular network by using an innovative dual-cell printing process supplemented with a core-shell nozzle. To this end, the authors used two bioinks, a collagen solution laden with Caco-2 cells (bioink-E) for the shell region and human umbilical vein endothelial cells (HUVECs; bioink-V) for the core region. Briefly, a layer of flat mesh structure is printed using bioink-V. Over this structure, the bioink-E is printed as a second layer and, lastly, a villus structure is vertically and simultaneously printed using a core-shell nozzle supplemented with the bioink E and V. The results presented showed that the cell-laden intestinal villi successfully mimicked the 3D geometry of human intestinal villi, and the cellular activity was also demonstrated, proving the efficacy and potential to be implemented of gut-on-a-chip platforms.

#### 3.2.9. Thrombosis-on-a-Chip

Thrombosis is the emergence of blood clots on the interior of blood vessels and it is the main cause of mortality. For this reason, realistic in vitro prototypes are needed to study this pathology.

Zhang and co-workers [53], by using sacrificial bioprinting, developed a thrombosis-on-a-chip. In this bioprinting technique, a mold is bioprinted using Pluronic as a sacrificial scaffold. Then, the mold was filled using GelMA solution and gelation was induced using photocrosslinking. Afterward, the Pluronic components were removed to obtain a hydrogel microchannel. Then, the hollow microchannels were coated with confluent endothelial layers, where the infusion of human whole blood occurred and induced the formation of thrombi and the continuous perfusion of an agent inductor of thrombolysis led to the dissolution of the non-fibrotic clots, showing the clinical relevance of the model. Moreover, it was unveiled that in the hydrogels containing fibroblasts and damaged endothelium, migration of fibroblasts into the clot, and subsequent deposition of type I collagen occurred, remodeling the fibrosis process in vivo. They showed a versatile platform for studying fibrosis, thrombolysis, and thrombosis, along with the interactions among the distinct components.

#### 3.2.10. Tumor Array-on-a-Chip

Xie et al. [54] developed a tumor array-on-a-chip by applying an electrohydrodynamic 3D printing with GelMA hydrogel droplets containing tumor cells produced by a high-voltage electric field force. Firstly, a high-voltage electric field is formed by connecting the metal plate of the 3D printer with a positive pole of the high-voltage power. Subsequently, a transparent conductive membrane was positioned on the metal plate, and GelMA bioink was introduced by the syringe pump on the 3D bioprinter. After finishing the array fabricating and crosslinking, the membrane was assembled with culturing chambers formed by s stainless steel and silicon interlayer, which can be recycled. The authors concluded that this tumor chip has a suitable microenvironment and containing tumor cell functionalization, and, thus, it can play a substantial role in the development of ground-breaking cancer therapies.

#### 3.2.11. Placenta-on-a-chip

Preeclampsia is considered part of the main causes of perinatal and maternal morbidity and mortality. This is characterized by elevated blood pressure and also a noteworthy quantity of protein present in the urine of pregnant women due to reduced trophoblast invasion of maternal spiral arteries. Nevertheless, there are few biomodels able to reproduce this pathology [23,55,57]. A promising model was proposed by Kuo and co-workers [55]. A bioprinted placenta model was developed to study how epidermal growth factor (EGF) affects the migratory conduct of human mesenchymal stem cells and trophoblast. A positive correlation between cell migration rates and EGF concentration was verified. Thus, EGF proved to be a potential therapeutic agent to treat preeclampsia. In addition, this model allows obtaining a significantly more realistic representation of the in vivo environment compared to that of current 2D models. By observing the prior discussed results, it can be seen that the liver-on-a-chip is the OoC device most developed and studied since the liver has a critical role in detoxification of blood and drug metabolism.

Regarding the 3D bioprinting approaches, despite some authors not providing the methodology used, it can be observed that there are many technologies found in the literature, including in-house developed 3D bioprinters, which is a quite interesting concept. Among the several approaches presented, the extrusion-based technique is the preferred one to fabricate more representative OoC platforms of in vivo conditions. In this approach, the continuous mechanic extrusion and rapid polymerization of hydrogel filaments of bioinks are done, and it can be used for depositing materials with a high cell concentration to accelerate tissue growth and formation, and also it allows the use of the broadest range of bioink viscosities [22,38]. Nevertheless, it should be noted that, besides the selection of the 3D bioprinting technology, the development of suitable and cytocompatible bioinks is of utmost importance for the successful bioprinting of living cells, since the mechanical and rheological properties of the hydrogel affect the stability of the bioprinting process and the activity of the encapsulated cells [49,58]. Among all biomaterials, gelatin-based bioinks are the most commonly applied, due to their capabilities of promoting cellular functionalization, creating suitable conditions for the differentiation and proliferation of cells, and rapid crosslinking [49,54,58]. However, studies have been conducted in order to optimize these bioinks [49,59]. For instance, Abudupataer et al. [49] found that the 5% GelMA bioinks have an excellent storage/loss modulus, indicating that the stability of bioink can be maintained for a long period and also that it has a slight influence on the viability of the cells in the printing process, due to a low level of shear stress during bioprinting.

Despite the outstanding properties of gelatin-based hydrogels, bioinks composed of other biomaterials have been proposed, as silk-based ink [51]. This new ink showed excellent properties for developing viable cardiac muscle tissues in vitro, while maintaining its functionality. Moreover, this bioink can be advantageous for obtaining other types of tissues. 

Furthermore, although the majority of studies identified addressed only one type of organ/tissue, the combination of multiple organs within a single microfluidic device, as a human-on-a-chip platform, is the future generation of OoCs devices, although it still is a challenging topic. However, this is of great importance for drug screening applications since toxic effects in secondary tissues are equally as important as the effects at the target site. If unobserved, these effects can lead to drug withdrawal from the market due to negative side effects [42].

### 3.3. New Approaches and Other Applications of 3D (Bio)Printing to Fabricate OoC Platforms without Specifying the Target Organ

As previously observed, the usage of 3D printing techniques to obtain 3D cell culture models has gained great interest. However, although several printing approaches were developed, novel approaches that allow printing of channels bearing tunable and user-defined complexity, morphology, and size are still needed, and efforts are being made in this direction [60].

Some researchers identified in the literature did not study OoC platforms by specifying the target organ; the results are summarized in Table 3.

Current bioprinting strategies present some limitations, in particular the high fidelity elaboration of complex 3D structures made of multiple bioinks capable of self-supporting without deformation [61]. In this sense, Rocca and colleagues [61] proposed a new method to extrude different bioinks from the same printhead for embedded extrusion bioprinting. To this end, three needles were linked together to originate a multi-material extrusion printhead. To test this methodology, the authors used Pluronic as the supporting hydrogel matrix due to its thermoresponsive and biocompatible properties. The selected bioink was alginate due to its low cost, fast physical gelation, and good biocompatibility. When the alginate bioink is extruded inside the Pluronic bath, a freeform structure can be obtained. Then, it is possible to remove the bioprinted structure from the supporting hydrogel to be used in further applications. This technique has great potential to improve the fabrication of more representative OoC platforms composed of intricate volumetric structures with different cell types, which cannot be accomplished with conventional layer-by-layer deposition. Moreover, in this way, it is not necessary to have different needles to print, being able to obtain the same result with only one, which is advantageous instead of the method presented by Lee et al. [46]. Patrício et al. [62] also explored the embedded 3D bioprinting technique with an alginate bioink. However, in this particular case, a different supporting material that consists of a continuous matrix of xanthan gum (XG), a cost-effective and widely available material with peculiar rheological properties, was proposed by the authors. One of the vital characteristics of XG is its pseudo-plasticity, in other words, its apparent viscosity declines with increasing shear force applied. After 3D printing, the XG matrix was removed by immersion in water. Additionally, the authors also demonstrated that, by modifying the XG with methacrylate groups, the XG can serve as the photocurable gel depository to set up the perfused cell-laden hydrogel pieces, which can be useful for the design of new OoC platforms. Another similar study was led by Ji et al. [60]. The research group proposed a novel sequential bioprinting approach that enables printing a sacrificial ink (Pluronic) within generally used photocurable hydrogels. For this purpose, photocurable hydrogels were printed layer-by-layer, with each layer being subjected to light to ensure the development of self-supporting layers. At the desired thickness, instantly afterwards the layer is printed, the sacrificial hydrogel is directly printed inside the viscous uncrosslinked layer. Afterward, the layer is put under light to support and confine the sacrificial hydrogel. The hydrogel is removed to form channels after entirely crosslinking the system. The results showed that endothelial cells adhered and formed confluent layers within these channels, and when incorporated into the photocurable ink formulations, stem cells remained highly viable inside the matrix hydrogels. Consequently, this approach has the potential to provide a valuable platform for fabricating vascularized tissues and studying cell behaviors.

A new approach was explored by Günther et al. [63]. They developed the first model of the standard micromachining device joining different lasers together with direct laser interference patterning (DLIP) and direct laser writing (DLW), a flexible and fast manufacturing process to obtain micro physiological systems. The proposed system has different print heads, inspection systems, laser sources, and processing optics on multiple axes, which enables the change and exact positioning to the workpiece throughout the process, making it possible to obtain 3D-printed components, as well as direct laser interference patterned surfaces for well-defined cell adhesion and product protection. Despite the features of this technology, further methods (i.e., temperature, beam profile) are intended to be integrated into the process. Another similar study was performed by Xiong and colleagues [64]. They used LIFT-based printing to produce heterogeneous cellular outlines in a lab-on-a-chip device. This technique is capable of creating any biological/cellular patterns and it can be applied to directly transfer cells and biomaterials to form customized patterns. As a demonstration of the present technique, the authors studied both the effect of cellular behavior and targeted drug delivery to cancer cells. For the first case, four different ECM formulations (collagen complemented with four different alginate concentrations) were tested, and it is found that less stiff dynamic culturing and ECM are preferred for the scattering of fibroblasts. For the second case, targeted delivery of cancer drugs to breast cancer cells was performed through different drug carriers, and it was discovered that the drug release rate from drug carriers affects the chemotherapy effect. Overall, the suggested laser printing-based method allowed the direct generation of heterogeneous cellular designs inside the lab-on-a-chip devices, improving the versatility and efficiency of drug screening and cell-related sensing.

Another interesting study was performed by Sonntag et al. [65]. The authors suggested an advanced lab-on-a-chip device for complex perfused 3D cell cultures. The device was composed of a multilayer elementary chip incorporating integrated pneumatically driven micropumps and valves and well-defined interfaces for an application-specific cell culture module. This cell culture module was printed by SLA and its surface was modified by laser micro structuring. To be noted, this module was intended for a direct interaction with robotic dispenser systems, which allows the combination of a microfluidic cell culture module with the direct organ printing of cells.

From the previous studies, it can be observed that embedded bioprinting has been commonly applied, mainly using Pluronic; however, efforts were made to improve the feasibility of this approach to fabricate 3D-printed cell culture platforms. An example is the XG, which showed to be a good candidate for the sacrificial matrix. Furthermore, as displayed in the first segment of the results, patterned surfaces for well-defined cell adhesion are also a very important issue that should be considered when 3D printing is used to obtain molds for casting methods. Although the current methods need to be further tested and improved, they are a good starting point for the creation of more complex and realistic OoC platforms.

## 4. Other Challenges in Organs-on-Chip Devices: Sensors Integration

For a standard laboratory practice, OoC platforms require an accurate control and monitoring of the cell metabolism and environment, as well as of the biomarkers released by the organ models into the feeding medium. Currently, this monitoring is mainly achieved by off-line post-analysis, which besides being time-consuming is prone to contamination and sample degradation. To overcome this limitation, micro(bio)sensors have been investigated to be incorporated into those platforms to allow for real-time, robust, and autonomous monitoring of the organ models. Compared with the standard methodologies in use, such as ELISA assays, biosensors allow for a reduction in the number of samples needed, have the ability to detect small quantities of biomarkers, and allow in situ and real-time analysis of biochemical parameters, with minor disturbances to the system. Nevertheless, some challenges still to be overcome for the successful integration of biosensors into OoC platforms are their miniaturization, the improvement of their biocompatibility, low limits of detection, flexibility, stretchability, and durability [9,66].

Among the sensors that have been developed to be included in OoC are pH and oxygen sensors [9]. For the former sensors, ion-selective field-effect transistors (ISFET), light addressable potentiometric sensors (LAPS), optical pH sensors, or metal oxide-based potentiometric sensors are typically used, the potentiometric sensors being preferred [67,68]. Regarding sensors for oxygen detection, optical or electrochemical detection methods are commonly used [67]. Although the latter are preferred, due to their fast assay time, easier integration, reproducibility, and robustness for online monitoring, the former is used in cases of low oxygen levels, since they do not need to be in physical or electrical contact with the solution and for continuous and long-term monitoring [9,67]. Other important development in biosensors for OoC are the electrochemical sensors that target specific cell products to monitor the cell activity, such as enzymes or enzyme byproducts. However, these sensors are mainly being developed as an external analytical module connected to the OoC. They require specific ligands to be immobilized on the surface of the working electrode. Consequently, their integration into the OoC platform, specificity, and long-term stability of the ligands are some of the main concerns of this technology, not achieved yet [9,69].

**Figure 4:**
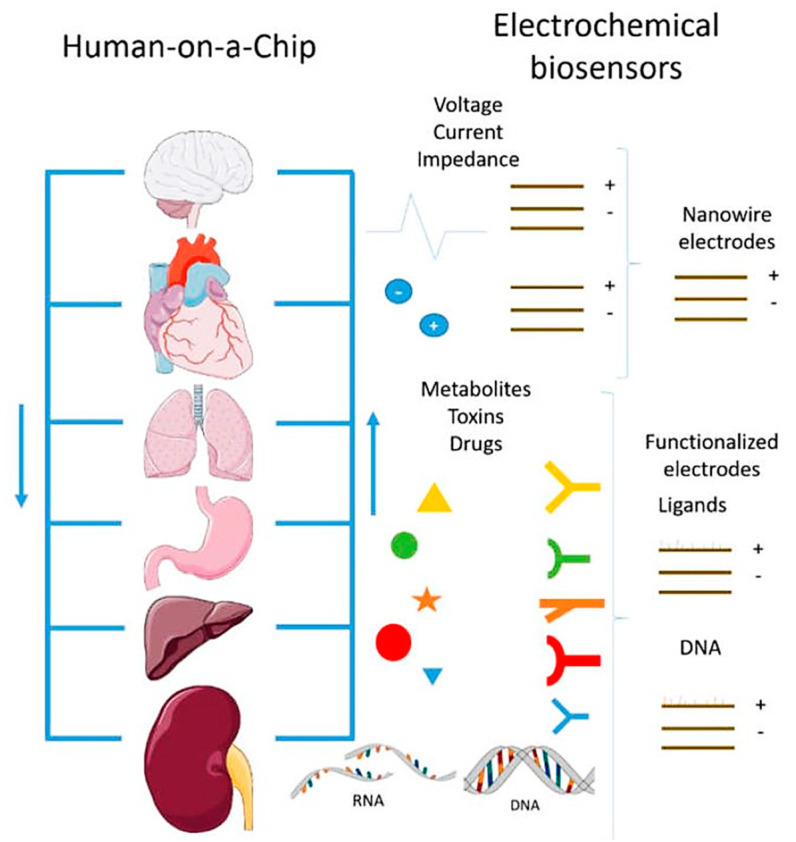
Representation of the biosensors for a human-on-a-chip platform. Reprinted from ref. [70].

In the near future, multi-organ-on-a-chip and, ultimately, human-on-a-chip platforms are expected to be developed. For that purpose, a platform with integrated biosensors will be a huge step towards the advance of OoC platforms, providing physiological metabolism parameters of the organ model, as presented at Figure 4 [70]. In this way, innovative experimental studies will be possible and as a result it will help to improve our understanding about the evolution of certain pathologies and how they affect the overall system [70]. A more comprehensive review on this topic can be found elsewhere [9]. 

## 5. Concluding Remarks and Future Perspectives

The selection of the appropriate fabrication method is an important step towards the achievement of highly reliable 3D culture models to investigate diseases, develop and test new drugs, and for drug-delivery strategies, among other medical applications. 3D (bio)printing techniques were selected and explored as fabrication methods in this systematic review owing to the many advantages of using these techniques, such as (i) the ability to accurately control the spatial distribution and layer-by-layer assembly of ECMs, cells, and other biomaterials; (ii) the generation of heterogeneous micro-organs; and (iii) a desired tissue-specific function and 3D cellular arrangement on a chip. 

From the overall results, it can be concluded that a wide variety of 3D printing techniques are available, and all of them, one way or another, are suitable for obtaining OoC, although it is not possible to establish the most appropriate one. Instead, it may be considered the combination of various printing methods and other techniques to complement the limitation of each method and maximize the advantages. For instance, attention must be paid when SLA or DLP techniques are used to obtain molds for OoC, because some residual monomers can remain on the top of the 3D-printed pieces, hampering PDMS polymerization and also compromising the cell behavior, and thus surface treatments may be needed.

Regarding the bioprinting techniques, the same observation is made: a wide variety of technologies are available, and they are appropriate to obtain realistic OoC. However, researchers are encouraged to improve and modify the existing techniques, by developing new nozzles or incorporating other components. For instance, an interesting improvement was presented by Rocca and colleagues [61] wherein a multi-material 3D printing approach was easily accomplished. By using only one nozzle, they were able to print distinct bioinks instead of using different nozzles. Other multi-material additive manufacturing methods have been explored in order to obtain a better cell seeding and cell adhesion efficiency, but this is still challenging, and extended knowledge is needed to develop more efficient multi-material 3D printers [22,71]. However, the most critical issue in the bioprinting process lies in the choice of the appropriate bioink with proper mechanical, rheological, and biological properties of the target tissues in order to protect and guarantee the cell viability during and after the printing process. Although gelatin-based hydrogels are the most commonly used, other formulations should be developed, for instance, by creating composite bioinks, combining the advantages of different materials, or making some modifications of the existing ones. An important finding in this field was presented by Mehrotra et al. [51], in which a non-mulberry silk fibroin was developed for engineering myocardial tissues. 

The combination of 3D printing techniques with the manufacture of OoC brings forth the possibility of forming heterogeneous structures using different cell types simultaneously to emulate the in vivo microenvironment. The printing of microelectronics on the chip contributed to the fabrication of microelectronic components that could not be achieved by traditional methods. Consequently, the development of hybrid bioprinters can allow the joint printing of the tissue and chip, making the fabrication of OoC a single-step process, viable for mass production. Overall, the technical breakthroughs in 3D printing ought to produce OoC with a high-throughput assay, high accuracy, high resolution, and multiple laboratory functions.

In summary, although great efforts for developing new and feasible 3D (bio)printing techniques have been made, wide-scale adoption and validation are still to be achieved. Through advances in 3D printing technologies, more physiologically relevant OoC models are expected and this will accelerate the commercialization of these models and their practical use in drug discovery to overcome several human diseases. Although the focus of this work lies in 3D (bio)printing, it should be mentioned that the variable “time” has also been integrated, giving rise to 4D bioprinting, where printed items (for example, responsive biocompatible materials or cells) are able to change their functionalities or shapes with time once an external stimulus is imposed [72].

## Figures and Tables

**Table 1:** 3D printing techniques used to fabricate OoC platforms.

Device	Printing Method	Application		Main Observations	Ref.
Vessel-on-a-chip	-	Produce molds with diverse forms of channels.	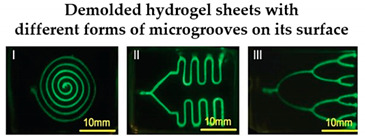 Reprinted with permission from ref. [32]. Copyright 2018 John Wiley and Sons.	A simple and cytocompatible approach was developed for fabricating hydrogel-based user-defined chips, suitable for the growth of organ or vascularized tissue models.	[32]
Lung cancer-on-a-chip	Inkjet	3D-printed chip holder and elastomeric microfluidic channels and microfluidic connectors for cell culture media routing on the higher part of the glass.	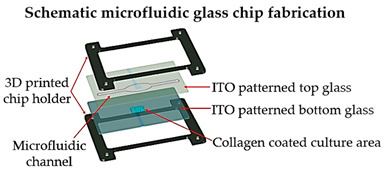 Reprinted with permission from ref. [33]. Copyright 2020 Elsevier.	This lung cancer-on-chip system, includes integrated biosensors for real-time monitoring of physiological events, can be used with any organ tissue or monolayer micro-tumor models for on-chip toxicity studies.	[33]
Metastasis-on-a-Chip	Plaster-based 3D printing	3D-printed inverted chamber/channel structures as molds.	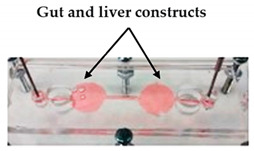 Reprinted with permission from ref. [4]. Copyright 2016 John Wiley and Sons.	This system supports some aspects of the phenomena of metastasis, allowing to study the translocation of metastatic tumor cells from the primary tissue site to the downstream tissue site.	[4]
Vessel-on-a-chip	Extrusion-based 3D printing	3D printing of channel prototypes with carbopol gel	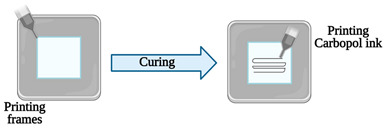	It is presented a highly affordable and practical approach in the manufacture of PDMS devices with closed fluid channels, which have great potential to reconstitute a human endothelium-on-a-chip	[15]
Kidney-on-a-chip	FDM	3D-printed template for conventional soft lithography fabrication of PDMS-based OoC	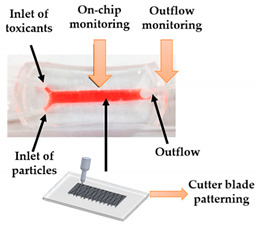 Reprinted with permission from ref. [34]. Copyright 2016 Elsevier.	It is demonstrated the application of a 3D-printed template and a common cutter machine to provide a simple and affordable fabrication of OoC.	[34]
Multi-Organ-On-a-Chip	Laser SLA with epoxy resin	Produce master models for the chambers and channels of the fluidic device.	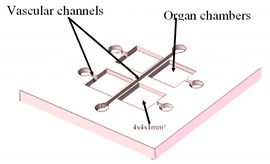 Reprinted from ref. [35].	This technology allows the design and rapid mass production of OoC devices.	[35]
Lung-on-a-chip	DLP	3D-printed molds to manufacture a chip model with an open well design and with lower and upper layers to mimic the human lung.	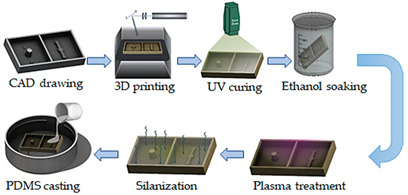 Reprinted from ref. [36].	The fabrication technique allows the chip to be fabricated in less than a day, and the molds can also be utilized for repeated PDMS casting. Therefore, the technique is robust, cost-effective, and simple.	[36]

SLA—stereolithography; FDM—fused deposition modelling; DLP—digital light processing.

**Table 2:** 3D bioprinting techniques used to fabricate OoC platforms.

OoC Platform	Printing Method	Schematic Representation	Cells Types	Bioink	Ref.
Nervous System-on-a-Chip	Micro-extrusion 3D printing strategies	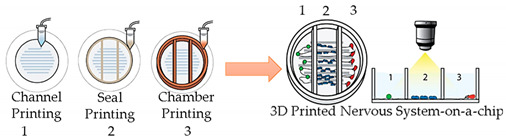 Reprinted with permission from ref. [40]. Copyright 2001 Royal Society of Chemistry.	Schwann cells, superior cervical ganglia and hippocampal neurons and epithelial cells	-	[40]
Central nervous system-on-a-chip	Magnetic bioprinting	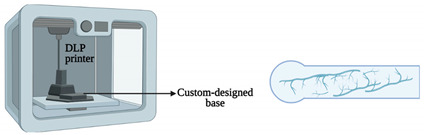	Spinal cord cells	Neural spheroids	[41]
Multi-tissue OoC with liver, heart and lung organoids	Microextrusion bioprinting	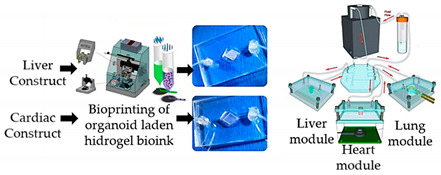 Reprinted from ref. [42].	Hepatocyte; stellate; Kupffer iPS; lung fibroblasts, epithelial, and endothelial cells.	Spherical organoids with HA-gelatin hydrogel (liver) and fibrin-gelatin bioink (cardiac).	[42]
3D vascularized tissue-on-a-chip	Microextrusion bioprinting	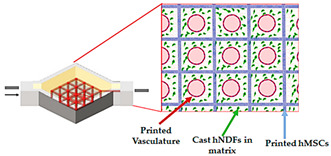 Reprinted from ref. [43].	hMSCs; hNDFs; HUVECs	Vascular ink (pluronic and thrombin) and cell-laden ink (gelatin–fibrin)	[43]
Liver-on-a-chip	Direct write bioprinter	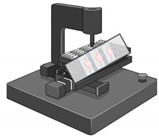	HepG2/C3A cells	Hepatic spheroids and GelMA	[44]
Liver-on-a-chip	Microextrusion bioprinting	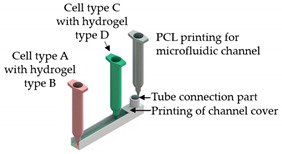 Reprinted from ref. [10].	HepG2; HUVECs.	Gelatin and liver dECM bioinks (collagen type 1)	[10]
Liver-on-a-chip	Microextrusion bioprinting	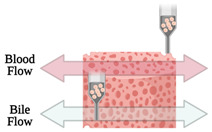	HepaRG and HUVECs	Gelatin and liver dECM bioinks (collagen type 1)	[45]
Liver Fibrosis-on-a-Chip	Microextrusion bioprinting	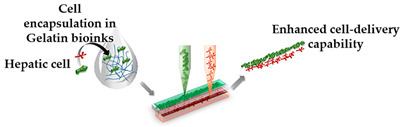 Reprinted with permission from ref. [46]. Copyright 2020 American Chemical Society.	HepaRG, HUVECs and hepatic stellate cells	Gelatin and liver dECM bioinks (collagen type 1)	[46]
Convoluted 3D renal proximal tubules-on-a-chip	Extrusion custom-designed, multi-material 3D bioprinter	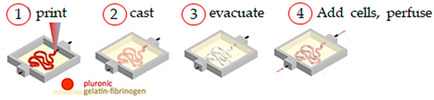 Reprinted from ref. [47].	PTECs-TERT1	Two-part silicone elastomer; Pluronic and thrombin.	[47]
Vessel-like structures-on-a-chip	Coaxial nozzle-assisted extrusion-based bioprinting	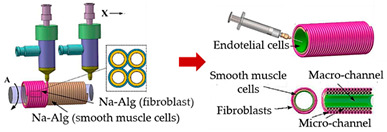 Reprinted with permission from ref. [48]. Copyright 2017 American Chemical Society	L929 fibroblasts; endothelial cells and smooth muscle cells	Cell-laden alginate filaments	[48]
Vessel-on-a-chip	-	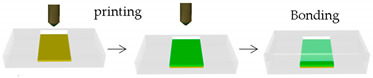	HAECs; HASMC and NIH/3 T3 fibroblast cell lines	GelMA	[49]
Heart-on-a-Chip	Direct write bioprinter with a customized coaxial nozzle	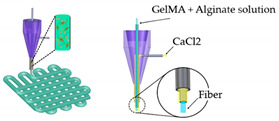 Reprinted with permission from ref. [50]. Copyright 2016 Elsevier	HUVECs	Alginate-GelMA	[50]
Myocardium-on-a-chip	Extrusion-based 3D bioprinting	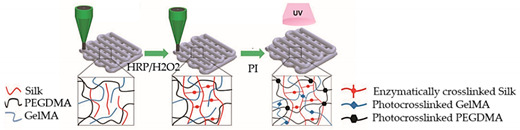 Reprinted with permission from ref. [51]. Copyright 2020 John Wiley and Sons.	hiPSC-CSs	Non-mulberry silk-based ink GelMA and PEGDMA	[51]
Gut-on-a-chip	Dual cell-printing system supplemented with a core-shell nozzle	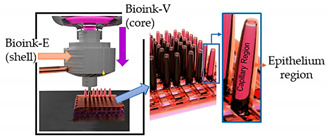 Reprinted with permission from ref. [52]. Copyright 2018 American Chemical Society.	Caco-2 cells and HUVECs	Cell-laden collagen bioinks	[52]
Thrombosis-on-a-chip	Embedded extrusion bioprinting	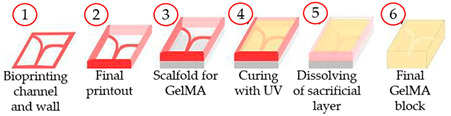 Reprinted with permission from ref. [53]. Copyright 2016 Royal Society of Chemistry.	HUVECs	GelMA	[53]
Tumor array-on-a-chip	On-demand array printing	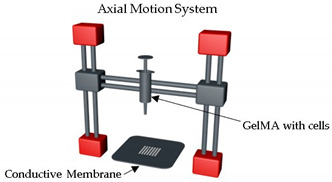	MDA-MB-231 breast tumor cells showed	GelMA	[54]
Placenta-on-a-chip	Extrusion-based 3D bioprinting	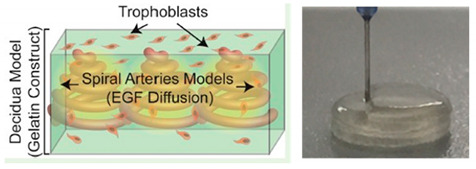 Reprinted with permission from ref. [55]. Copyright 2016 American Chemical Society.	Human placental cell line and hMSCs	GelMA	[55]

iPS—induced pluripotent stem cells; HA—hyaluronic acid; hMSCs—human mesenchymal stem cells; hNDFs—human neonatal dermal fibroblasts; HUVECs—human umbilical vein endothelial cells; HepG2—human hepatocellular carcinoma; HepaRG—terminally differentiated human hepatocellular carcinoma cells; dECM—decellularized extracellular matrix; PTECs—proximal tubule epithelial cells; TERT1—human telomerase reverse transcriptase; HAECs—primary human aortic endothelial cells; HASMC—human aortic smooth muscle cell line CRL1999; GelMA—gelatin methacryloyl; hiPSC-CSs—human-induced pluripotent stem cell-derived cardiac spheroids; PEGDMA—polyethylene glycol dimethacrylate; BMECs—human bone marrow endothelial cells.

**Table 3:** Novel approaches of 3D bioprinting/printing techniques suitable for the manufacturing of OoC platforms.

3D (Bio)Printing Technology	Schematic Representation	Ref.
Embedded extrusion bioprinting	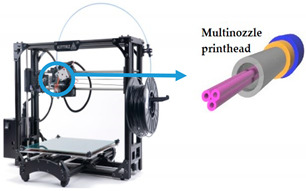 Reprinted from ref. [61].	[61]
Embedded extrusion bioprinting	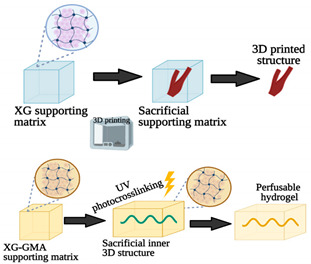	[62]
Embedded extrusion bioprinting	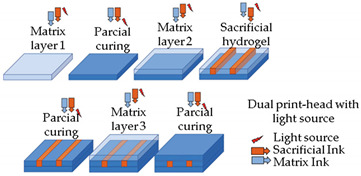 Reprinted with permission from ref. [60]. Copyright 2019 Elsevier.	[60]
DLW and DLIP	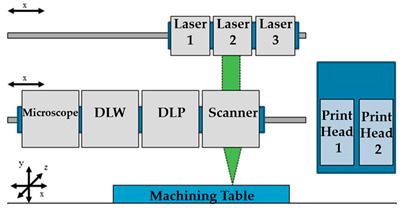 Reprinted from ref. [63].	[63]
LIFT printing	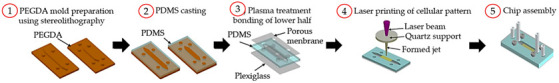 Reprinted with permission from ref. [64]. Copyright 2019 Royal Society of Chemistry.	[64]
SLA and Bioprinting (*BioScaffolder*)	^ 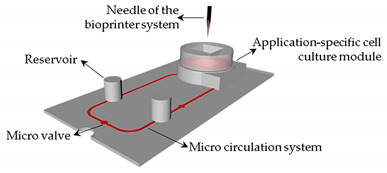 ^	[65]

DLW—direct laser writing; LIFT—laser-induced forward transfer; DLIP—direct laser interference patterning.

## Data Availability

Not applicable.
